# EASILY ACCESSIBLE EVALUATIVE INFORMATION COULD FACILITATE OLDER ADULTS’ (BUT NOT ALL) TRUSTWORTHINESS JUDGEMENT

**DOI:** 10.1093/geroni/igz038.3144

**Published:** 2019-11-08

**Authors:** Linlin Chen, Xin Zhang

**Affiliations:** 1 School of Psychological and Cognitive Sciences, Peking University, Beijing, China; 2 Peking University, Beijing, China

## Abstract

Facial appearance served great function in social interactions, especially for older adults in making trustworthiness judgements. Previous literatures have consistently shown that when making trustworthiness judgements older adults tended to rely more on facial cues rather than behaviors, due to declines in cognition. However, one question remains unsolved, whether older adults could make accurate trustworthiness judgements if evaluative information (with minimal memory load) is easily accessible. Sixty younger adults (YAs) and sixty older adults (OAs) were recruited, and asked to make investment decisions for different brokers in ninety-six trials. In each trial, brokers’ facial appearance (trustworthy and untrustworthy looking) and different behavioral evaluative information (good: Ninety percent positive evaluations, neutral: Fifty percent positive evaluations, bad: Ninety percent negative evaluations) were displayed simultaneously on screen to facilitate investment decisions. Brokers’ facial appearances and behaviors were set to be independent to each other. The results indicated that YAs’ and the majority of OAs’ proportions of correct investment increase, gradually reaching a stable high correction rate, although OAs needed more trials than did YAs. The findings extended prior work by suggesting that both OAs and YAs had similar abilities to distinguish different brokers according to easily accessible evaluative information. However, and surprisingly, a small subgroup of OAs (with low economic status) still had a lower correction rate even after ninety-six trials, suggesting that they could not distinguish brokers based on their evaluations at all, who might be at risk for fraud.

